# A retrospective cohort study comparing different fixation methods for the MiniMizer Extra adjustable gastric band

**DOI:** 10.20452/wiitm.2025.17937

**Published:** 2025-03-24

**Authors:** Žygimantas Juodeikis, Gintautas Brimas

**Affiliations:** Clinic of Gastroenterology, Nephro‑Urology and Surgery, Institute of Clinical Medicine, Vilnius University, Vilnius, Lithuania

**Keywords:** adjustable gastric banding, bariatric and metabolic surgery, obesity

## Abstract

**INTRODUCTION:**

Two decades ago, laparoscopic adjustable gastric banding was a leading bariatric sur‑ gery. However, its popularity has declined, with sleeve gastrectomy becoming the predominant choice. The MiniMizer Extra band used in our clinic from 2008 to 2020 was associated with band erosion primarily at its lower edge. In 2014, we started using a modified band fixation technique by placing sutures only on the upper part of the band.

**AIM:**

The aim of this study was to compare 2 different fixation techniques for the MiniMizer Extra adjustable gastric band to identify any potential differences in outcomes.

**MATERIALS AND METHODS:**

In this study, we compared 54 patients who underwent adjustable gastric banding with the MiniMizer Extra band between January 1, 2009, and January 31, 2010, with a group of 54 patients who were subjected to the procedure between January 1, 2014, and January 31, 2019, using a different band fixation method.

**RESULTS:**

Weight loss results significantly favored the modified fixation group, with an average total weight loss of 24.2%. The overall complication rate was 12% and was significantly higher in the original fixation group. Complications included 6 cases of band erosion, 4 port‑related issues, 1 case of band slippage, and 2 cases of band intolerance.

**CONCLUSIONS:**

The modified fixation group demonstrated improved weight loss results with fewer com‑ plications, suggesting a potential advantage in safety and efficacy of the modified technique.

## INTRODUCTION

Two decades ago, laparoscopic adjustable gastric banding (LAGB) was a widely accepted and standardized restrictive procedure, serving as the primary type of bariatric surgery. However, its popularity has since declined, having been largely replaced by sleeve gastrectomy.^1^ During this period, various adjustable gastric bands were introduced, each with distinct features in terms of design, filling capacity, internal pressure, and fixation techniques.^2^ These differences are thought to influence long‑term outcomes.^3^^,^^4^ While many studies have compared gastric bands, the majority have focused on the Swedish adjust‑ able gastric band (SAGB) and LAP‑BAND systems. 

In our previous study involving 54 patients with the MiniMizer Extra adjustable gastric band (Bariatric Solutions GmbH, Münchenstein, Switzerland), 5 cases of band erosion were observed, all located at the lower part of the band.^4^ The authors hypothesized that the pressure exerted by the lower edge of the band on the stomach wall played a critical role in erosion, likely increasing as the portion of the stomach above the band filled. Consequently, starting in 2014, the band fixation technique was modified, with sutures applied only to the upper part of the band. This prompted a study comparing 2 different fixation techniques for the MiniMizer Extra adjustable gastric band to identify any potential differences in outcomes.

## MATERIALS AND METHODS

We conducted a retrospective cohort study comparing 54 patients from a prospective randomized trial who underwent adjustable gastric banding with the MiniMizer Extra band between January 1, 2009, and January 31, 2010 [4] with a group of patients who were subjected to the procedure be‑ tween January 1, 2014, and January 30, 2019, using a modified band fixation method.

**TABLE 1:** Baseline characteristics

Parameter		All patients (n = 108)	Original MiniMizer group (n = 54)	Modified fixation group (n = 54)	P value
Age, y		45.4 (10.1)	45.8 (11.9)	45 (12.3)	0.73
Sex, n (%)	Women	72 (66.6)	38 (70.4)	34 (62.9)	0.54
	Men	36 (33.3)	16 (29.6)	20 (37.1)	
Body weight, kg		135.8 (23.8)	133.8 (24)	138 (21.2)	0.37
BMI, kg/m2		46.7 (6.9)	46.5 (6.7)	46.9 (9.3)	0.74

Values are presented as mean (SD) unless stated otherwise.

Abbreviations: BMI, body mass index

**TABLE 2:** Weight parameters 5 years after laparoscopic adjustable gastric banding

Weight variable	All patients	Original MiniMizer group	Modified fixation group	*P* value
Weight, kg	100.8 (23.9)	104.8 (26.6)	96.4 (24.0)	0.052
BMI, kg/m2	34.5 (7.2)	36.0 (7.8)	32.8 (7.7)	0.01
BMIL, kg/m2	12.1 (7.3)	10.6 (6.6)	14.1 (9.3)	0.004
%TWL, %	25.1 (13.3)	22.6 (13)	28.6 (15.9)	0.005
%EWL, %	56.1 (27.3)	50.3 (27.6)	63.6 (31.1)	0.003

**FIGURE 1:**
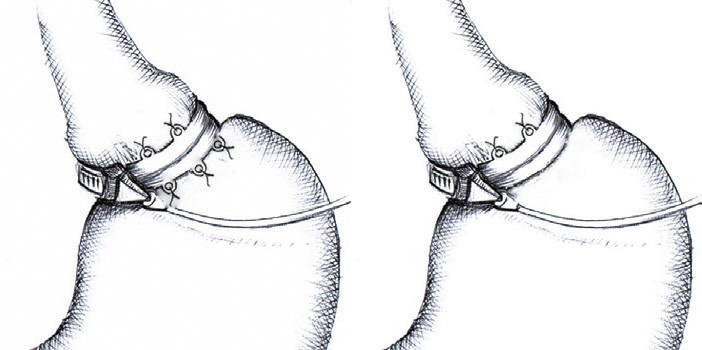
Fixation techniques: A – original; B – modified

Change Image

Patients were eligible if they were between 18 and 70 years old, with a body mass index (BMI) greater than 40 kg/m2 or between 35 and 40 kg/m2 accompanied by obesity‑related comorbidities. Exclusion criteria comprised a history of previous bariatric surgery, pregnancy, or any contraindications to laparoscopic surgery. The patients in the modified fixation group were contacted by phone, and those who consented to undergo upper gastrointestinal (GI) endoscopy were included in the study. Patients’ weight measurements were taken during the follow‑up visits. Complication data were collected from medical records and the postoperative follow‑up visits.

BMI loss (BMIL) was defined as the difference between the preoperative BMI and the BMI at follow‑up. Total weight loss percentage (%TWL) was calculated using the following formula: (initial weight − follow‑up weight)/initial weight×100. Excess weight loss percentage (%EWL) was de‑ fined as follows: (initial weight − follow‑up weight)/(initial weight−ideal weight) × 100, where the ideal weight corresponded to the BMI of 25 kg/m².

### Ethics

This study was conducted in accordance with the Declaration of Helsinki and was ap‑ proved by the Vilnius Regional Biomedical Research Ethics Committee on March 23, 2021 (2021/3‑1319‑797). Informed consent was obtained from all the patients before participation in the study. Data collection and analysis were performed with strict adherence to patient confidentiality and ethical guidelines.

### Surgical technique

Laparoscopic gastric banding was performed using the pars flaccida technique in both groups. In the original MiniMizer Extra group, the retaining loops were attached direct‑ ly to the anterior gastric wall with 5 interrupted 2–0 silk sutures: 2 on the upper and 3 on the low‑ er edge. In the modified fixation group, only the 2 upper retaining loops were used FIGURE 1. The access port was implanted subcutaneously and secured to the left rectus fascia with interrupted nonabsorbable sutures. All procedures were performed by a single surgeon with prior experience in over 600 LAGB operations.

### Statistical analysis

The IBM SPSS Statistics for Windows package, version 21.0 (IBM Corp., Armonk, New York, United States) was used for statistical analysis. Categorical variables were com‑ pared using the Pearson χ2 or Fisher exact test, and the t test or Mann–Whitney 2‑sample tests was used for continuous variables depending on distribution. A P value of less than 0.05 was con‑ sidered significant.

## RESULTS

A total of 99 patients who underwent adjustable gastric banding using the modified fixa‑ tion technique between January 1, 2016, and Jan‑ uary 30, 2019, were identified. Of these, 54 pa‑ tients consented to a follow‑up visit to be subject‑ ed to upper GI endoscopy. The outcomes of these patients were compared with those of 54 patients who underwent adjustable gastric banding with the MiniMizer Extra band using the convention‑ al fixation method between January 1, 2009, and January 31, 2010.

The baseline characteristics of both groups are presented in TABLE 1. Over a mean (SD) follow‑up period of 5.5 (2.89) years, the mean (SD) number of band adjustments was 4.7 (3.1) in the modified fixation group and 3.9 (3.29) in the original fixa‑ tion group (P = 0.02).

**TABLE 3:** Complications 5 years after laparoscopic adjustable gastric banding

Adverse event	Total	Original MiniMizer group	Modified fixation group	*P* value
Band erosion	6 (5.5)	5 (9.2)	1 (1.8)	0.21
Band slippage	1 (0.9)	1 (1.8)	0	1
Band intolerance	2 (1.8)	2 (3.7)	0	0.5
Port related	4 (3.7)	3 (5.5)	1 (1.8)	0.62
Total	13 (12)	11 (20.3)	2 (3.7)	0.007

Values are presented as number (percentage) of events.

In the original fixation group, 8 patients were lost to follow‑up after 5 years. One patient died, 5 had their bands removed (3 due to band erosion and 2 due to psychological intolerance and insufficient weight loss), while 2 patients were unreachable.

Weight loss parameters at 5 years are outlined in TABLE 2, with an average %TWL of 25.1%. Significant differences in favor of the modified fixation group were observed in BMIL, %TWL, and %EWL. The overall complication rate at 5 years was 12%, with fewer complications in the modified fixation group (P = 0.007).

Complications included 6 cases of band erosion, 4 port‑related issues, 1 case of band slippage, and 2 cases of band intolerance. Port‑related complications comprised 3 port‑site infections and 1 port inversion, none of which were associated with band erosion. All complications are listed in TABLE 3. All 6 cases of band erosion required removal (1 in the modified fixation group and 5 in the original fixation group; P = 0.09). Band slip‑ page was managed by laparoscopic repositioning. All 4 patients with port‑related complications required port reimplantation.

## DISCUSSION 

Various adjustable gastric bands are used globally, differing in terms of design, filling volume, internal pressure, and fixation mechanisms. These variations could potentially impact long‑term outcomes. The MiniMizer Extra adjustable gastric band is a low‑volume, high‑pressure system. This design may exert more localized pressure on the gastric wall, leading to a higher risk of band erosion and slippage. In contrast to other systems, the MiniMizer Ex‑ tra band is anchored to the gastric wall using re‑ taining loops, which eliminates the need for pli‑ cation during fixation. Moreover, the band is de‑ signed with a 2‑phase closure mechanism that allows for precise adjustment of the inner diameter based on intraoperative assessments to optimize fit and function.

Our findings align with previously published literature in terms of %TWL and %EWL, with the average total weight loss in this study being 25.1%, which is consistent with multiple studies evaluating adjustable gastric bands. However, the %EWL after 5 years was higher in the modified fixation group (63.6%) compared with the original fixation group (50.3%). This difference is noteworthy, as other studies, such as the one conducted by O’Brien et al,5 have reported mean %EWLs ranging between 48% and 57%. Also, bet‑ ter weight loss results in the modified fixation group may be partially attributed to the higher number of band adjustment visits. This factor was also higher in this group (P = 0.02).

The complication rates observed in our study (12%) are also within the range reported in high‑volume centers, where long‑term complication rates vary between 6% and 25%,^2^^,^^6^ the most com‑ mon being band slippage, band erosion, and port‑related issues.^6^^,^^7^ These findings suggest that while our results are in line with global data, the modified fixation technique may offer superior weight loss outcomes without increasing complication rates. Further investigations are warranted.

The incidence of band erosion with the pars flaccida technique ranged from 0% to 1.6%.^8^ The exact mechanism behind this complication remains unclear. Proposed etiological factors include intraoperative gastric wall injury, band infection, and overfilling during band adjustments.^9^ One potential explanation for the lower rate of band erosion in the modified fixation group may be related to the altered pressure dynamics resulting from suturing only the upper part of the band. Band erosion is a serious complication associated with adjustable gastric bands, and its exact cause remains unclear. However, it is hypothesized that excessive pressure exerted by the band on the gastric wall, particularly at the lower edge, may contribute to erosion.^9^ In the modified fixation technique, reducing the contact area between the band and the gastric wall by limiting sutures to the upper part could theoretically minimize this pressure and reduce the risk of erosion. This study sup‑ ports that hypothesis, as there was only 1 case of erosion in the modified fixation group, com‑ pared with 5 in the original group, though the dif‑ ference was not significant. Further research is needed to explore the underlying physiological mechanisms and assess whether the modified fix‑ ation technique reduces erosion risk over more extended periods.

In the original fixation group, of the 5 eroded bands, 3 were removed and the remaining 2 patients were asymptomatic with partial erosion (<30% of the band circumference). Thus, the planned endoscopic removal was postponed. The single patient with band erosion in the modified fixation group underwent successful endoscopic removal. These findings align with previously published data.10,11 There is no consensus on the best method of band removal.12 In our opinion, an eroded band should be removed using the least invasive method, that is upper GI endoscopy. In our study, 2 bands were removed endoscopically and 2 laparoscopically. One laparoscopic removal was required due to an intra‑abdominal abscess, while the other followed a failed endoscopic attempt.

Complications related to the ports reportedly occur in 4% to 20% of patients.^13^^,^^14^ Although these complications are generally considered minor, most require surgical intervention. In our series, 4 patients (3.7%) experienced port‑related complications, including 3 cases of port‑site infection and 1 case of port rotation, with no significant difference between the 2 groups.

This study was facilitated by its longitudinal perspective, evaluating both types of band fixation in a direct manner across time. Including both clinical and complication results renders a valid investigation of the safety and effective‑ ness of each method. Also, all procedures were performed by a single highly experienced surgeon who ensured the uniformity of the surgical technique.

Although we provided follow‑up rate data for the original fixation group, the study design inherently prevented loss to follow‑up in the modified fixation group, which may introduce potential bias.

There is also a possibility of patient selection bias, as only 54 out of 99 patients who under‑ went the modified fixation technique consented to a follow‑up visit with upper GI endoscopy. It is possible that patients who declined follow‑up had less favorable outcomes, which could have influenced the overall findings.

It is generally proven that in the long term, LAGB is associated with inferior weight loss results and more complications compared with sleeve gastrectomy and gastric bypass.^3^^,^^15^ There‑ fore, the findings of this study are clinically relevant, particularly for centers that still perform LAGB. While the use of adjustable gastric bands has declined globally in favor of procedures such as sleeve gastrectomy, our results suggest that certain fixation methods may enhance the safety and efficacy of LAGB. The significantly better weight loss outcomes observed in the modified fixation group, without an increase in complication rates, indicate that this technique could be preferred in centers that continue to use the MiniMizer Extra or similar bands. Nevertheless, due to the limitations of this study, which include small sample size and retrospective design, larger and comprehensive prospective research should be carried out.

## CONCLUSIONS

The modified fixation technique for the MiniMizer Extra adjustable gastric band demonstrated improved weight loss outcomes and a lower complication rate, as compared with the original fixation method. The patients in the modified fixation group achieved greater total weight loss, %EWL, and BMIL, which may be at‑ tributed to both the altered fixation method and a higher frequency of band adjustments. Addition‑ ally, the lower incidence of complications, particularly band erosion, suggests that this technique

may enhance the long‑term safety and durability of the procedure.
